# Supplementary material for: MetaRibo-Seq measures translation in microbiomes
Source: Nat Commun. 2020 Jun 29;11:3268. doi: 10.1038/s41467-020-17081-z (PMC7324362; doi:10.1038/s41467-020-17081-z)
Supplement: Supplementary file 10 — Supplementary Data 7 [file 41467_2020_17081_MOESM10_ESM.zip › File2/Confidence_High_Taxonomy/155259_out.krona.html]

Javascript must be enabled to view this page.

members
magnitude
magnitudeUnassigned
count
unassigned
taxon
rank

155259\_out

172

2
172
superkingdom

4

SRS098827\_contig\_number\_4520SRS1041031\_contig\_number\_16099SRS1041037\_contig\_number\_contig-100\_9119.9120SRS893373\_contig\_number\_contig-100\_1647.146971
171
1239
phylum

186801
167
class

186802
order
25

SRS013687\_contig\_number\_35105SRS013965\_contig\_number\_42032SRS015431\_contig\_number\_20728SRS018427\_contig\_number\_26010SRS019068\_contig\_number\_112827SRS019286\_contig\_number\_2287SRS047014\_contig\_number\_contig-100\_7954.188154SRS048870\_contig\_number\_contig-100\_2906.130783SRS051882\_contig\_number\_22675SRS053356\_contig\_number\_contig-100\_2542.259829SRS054956\_contig\_number\_16436SRS063127\_contig\_number\_2243SRS064276\_contig\_number\_contig-100\_3217.227939SRS075341\_contig\_number\_contig-100\_1793.1793SRS077730\_contig\_number\_contig-100\_1365.1365SRS077849\_contig\_number\_contig-100\_6298.6298SRS101433\_contig\_number\_contig-100\_13210.53873SRS1041033\_contig\_number\_14582SRS143181\_contig\_number\_7987SRS144714\_contig\_number\_contig-100\_6576.140953SRS147139\_contig\_number\_contig-100\_1175.238929SRS147377\_contig\_number\_4632SRS147766\_contig\_number\_contig-100\_9109.108649SRS147977\_contig\_number\_contig-100\_2908.90277SRS148970\_contig\_number\_contig-100\_4394.33018
167

family
186803
108

SRS048060\_contig\_number\_13265SRS146812\_contig\_number\_63803SRS148319\_contig\_number\_7282
3

genus
2
2569097

species
2
39488

SRS1055022\_contig\_number\_26310SRS143780\_contig\_number\_33372

33042
genus
1


SRS045826\_contig\_number\_1560
33043
1
species

genus
1
2005359

39482

SRS015794\_contig\_number\_13268
species
1

12
genus
1407607

species
12
1150298

SRS023526\_contig\_number\_31901SRS024663\_contig\_number\_contig-100\_3054.42461SRS049712\_contig\_number\_19739SRS052697\_contig\_number\_46816SRS054905\_contig\_number\_26379SRS056273\_contig\_number\_contig-100\_464.203824SRS058070\_contig\_number\_29151SRS075078\_contig\_number\_26231SRS077086\_contig\_number\_11418SRS077502\_contig\_number\_contig-100\_2070.105666SRS142503\_contig\_number\_33965SRS143876\_contig\_number\_39576


SRS019178\_contig\_number\_2230SRS043667\_contig\_number\_6483SRS051610\_contig\_number\_2806SRS893230\_contig\_number\_12514SRS893231\_contig\_number\_contig-100\_544.50152
39491
species
5

58
2

SRS055982\_contig\_number\_contig-100\_796.120167SRS097958\_contig\_number\_contig-100\_3103.148299
genus
572511

species
30
33039

SRS011302\_contig\_number\_30912SRS013965\_contig\_number\_41928SRS015065\_contig\_number\_1018SRS015578\_contig\_number\_29898SRS016335\_contig\_number\_contig-100\_496.259563SRS016954\_contig\_number\_11597SRS017191\_contig\_number\_16981SRS017307\_contig\_number\_2508SRS020328\_contig\_number\_39404SRS023346\_contig\_number\_1084SRS023715\_contig\_number\_17436SRS042966\_contig\_number\_16877SRS043701\_contig\_number\_14056SRS043768\_contig\_number\_contig-100\_584.185900SRS044535\_contig\_number\_25664SRS063813\_contig\_number\_contig-100\_648.39883SRS064276\_contig\_number\_contig-100\_4057.228779SRS077127\_contig\_number\_4358SRS1041091\_contig\_number\_22892SRS1041138\_contig\_number\_contig-100\_4060.4061SRS1055049\_contig\_number\_17402SRS140645\_contig\_number\_2138SRS142599\_contig\_number\_contig-100\_303.188458SRS142890\_contig\_number\_41496SRS142980\_contig\_number\_7149SRS146764\_contig\_number\_contig-100\_150.206515SRS147977\_contig\_number\_13918SRS148159\_contig\_number\_contig-100\_6576.281678SRS148511\_contig\_number\_20807SRS893358\_contig\_number\_10353

3
species

SRS019910\_contig\_number\_contig-100\_948.137130SRS022137\_contig\_number\_17422SRS1054716\_contig\_number\_contig-100\_137.90391
2292970

40520

SRS019685\_contig\_number\_contig-100\_397.231281SRS022713\_contig\_number\_contig-100\_215.57866SRS023583\_contig\_number\_15890SRS057478\_contig\_number\_8572
4
species

species
3
1520805

SRS016095\_contig\_number\_30664SRS054590\_contig\_number\_contig-100\_598.29607SRS142712\_contig\_number\_contig-100\_1264.45315

species
8

SRS016495\_contig\_number\_contig-100\_433.122231SRS020869\_contig\_number\_38208SRS023914\_contig\_number\_22253SRS024388\_contig\_number\_contig-100\_240.98512SRS048164\_contig\_number\_contig-100\_2235.179704SRS057717\_contig\_number\_4759SRS075398\_contig\_number\_contig-100\_4353.4354SRS144506\_contig\_number\_14200
33038

7
species

SRS014459\_contig\_number\_33192SRS014459\_contig\_number\_38139SRS018936\_contig\_number\_12785SRS077392\_contig\_number\_43980SRS148721\_contig\_number\_50925SRS893270\_contig\_number\_23134SRS893369\_contig\_number\_12042
765821

species
1
2292964

SRS075963\_contig\_number\_contig-100\_396.396

189330
7
genus

species
3
39486

SRS011134\_contig\_number\_33930SRS019397\_contig\_number\_12845SRS147919\_contig\_number\_20999

4
species

SRS013800\_contig\_number\_26056SRS014923\_contig\_number\_46588SRS022609\_contig\_number\_46331SRS104485\_contig\_number\_contig-100\_675.48359
88431

841
genus
19

1
species

SRS149181\_contig\_number\_6508
301301

species
1
166486

SRS1055034\_contig\_number\_12467


SRS1055076\_contig\_number\_12263
1262943
species
1

1638781

SRS013940\_contig\_number\_19997SRS014948\_contig\_number\_21678SRS017433\_contig\_number\_15992SRS017916\_contig\_number\_12756SRS018351\_contig\_number\_contig-100\_3333.119757SRS018623\_contig\_number\_14778SRS019030\_contig\_number\_15704SRS019161\_contig\_number\_27780SRS021219\_contig\_number\_10092SRS024075\_contig\_number\_13095SRS050998\_contig\_number\_228SRS104311\_contig\_number\_45931SRS104636\_contig\_number\_31254SRS144362\_contig\_number\_39407SRS147557\_contig\_number\_contig-100\_16964.147233SRS149879\_contig\_number\_27443
16
species

1
species
1897042

SRS078242\_contig\_number\_contig-100\_758.200360

31979
2
family

1485
genus
2

species
1

SRS014235\_contig\_number\_27515
1262845

species
1
2292006

SRS054590\_contig\_number\_1791

3
family
186804

3
genus
1870884

1496

SRS053214\_contig\_number\_14637SRS065176\_contig\_number\_contig-100\_302.108793SRS077641\_contig\_number\_23048
species
3

family
1
186806


SRS077194\_contig\_number\_contig-100\_1359.193379
1730
genus
1

541000
27
family

25

SRS011084\_contig\_number\_contig-100\_1222.205113SRS015217\_contig\_number\_15296SRS024492\_contig\_number\_20129SRS046717\_contig\_number\_contig-100\_714.715SRS063518\_contig\_number\_39991SRS097889\_contig\_number\_41777SRS1041118\_contig\_number\_16776SRS1041142\_contig\_number\_contig-100\_78.62305SRS1055038\_contig\_number\_6207
9
genus
1263


SRS024435\_contig\_number\_41318SRS148424\_contig\_number\_31802
2292069
2
species

11
species
657323

SRS015190\_contig\_number\_contig-100\_480.101413SRS017247\_contig\_number\_8637SRS043411\_contig\_number\_18196SRS050422\_contig\_number\_contig-100\_462.202366SRS050752\_contig\_number\_contig-100\_4182.184861SRS063040\_contig\_number\_contig-100\_2186.267176SRS076929\_contig\_number\_27518SRS077454\_contig\_number\_19903SRS100021\_contig\_number\_contig-100\_12720.12720SRS105153\_contig\_number\_contig-100\_2160.231555SRS1054691\_contig\_number\_564

3
species

SRS015782\_contig\_number\_41695SRS062701\_contig\_number\_5897SRS892882\_contig\_number\_15174
165186

2
genus
216851

species
2

SRS105082\_contig\_number\_23441SRS893378\_contig\_number\_23508
853

phylum
1
976

200643
1
class

171549
order
1

2005525
1
family

375288
1
genus


SRS893285\_contig\_number\_2639
823
species
1
